# Exploring *Paenibacillus terrae* B6a as a sustainable biocontrol agent for *Fusarium proliferatum*

**DOI:** 10.3389/fmicb.2025.1547571

**Published:** 2025-03-03

**Authors:** Enriquay Smith, Augustine Innalegwu Daniel, Chelsey Smith, Stacey Fisher, Mbukeni Nkomo, Marshall Keyster, Ashwil Klein

**Affiliations:** ^1^Plant Omics Laboratory, Department of Biotechnology, Faculty of Natural Sciences, University of the Western Cape, Bellville, South Africa; ^2^Department of Biochemistry, School of Life Sciences, Federal University of Technology, Minna, Nigeria; ^3^Environmental Biotechnology Laboratory, Department of Biotechnology, Faculty of Natural Sciences, University of the Western Cape, Bellville, South Africa; ^4^Plant Biotechnology Laboratory, School of Life Sciences, University of KwaZulu Natal, Westville Campus, Westville, South Africa

**Keywords:** agriculture, bio-fungicide, food security, *Fusarium proliferatum*, biological control, phytopathogens

## Abstract

The reliance on chemical fungicides for crop protection has raised environmental and health concerns, prompting the need for sustainable and eco-friendly alternatives. Biological control, using antagonistic microorganisms like *Paenibacillus terrae* B6a, offers an eco-friendly approach to managing disease causing phytopathogens. The objective of the study was to assess the efficacy of *P. terrae* B6a as a biocontrol agent against *Fusarium proliferatum* PPRI 31301, focusing on its *in vitro* antagonistic activity, its impact on fungal morphology and enzymatic content, and its ability to mitigate pathogen-induced stress in maize plants. *In vitro* antagonistic activity of B6a against *F. proliferatum* was carried out using standard protocol. *In planta* assay was carried out by bio-priming of maize seeds with 1 × 10^6^ CFU/mL of B6a and infected with *F. proliferatum* for 7 days. Biochemical, enzymatic and antioxidants activities of bio-primed maize roots under *F. proliferatum* infection was carried out using spectrophotometric methods. *In vitro* antagonistic assays using dual culture and intracellular crude metabolites inhibited 70.15 and 71.64%, respectively, of *F. proliferatum*. Furthermore, B6a altered the morphology and mycelia structure of *F*. *proliferatum* under High resolution scanning electron microscopy (HR-SEM). This was supported by an increase (*p* < 0.05) in the chitin contents (48.03%) and a decrease (*p* < 0.05) in the extracellular polysaccharide content (48.99%) and endo-β-1,4-glucanase activity (42.32%). The infection of maize seeds with *F. proliferatum* resulted in a significant decrease (*p* < 0.05) in root lengths (37%). Relative to the control and the infected seeds, bio-priming with B6a shows a significant increase (*p* < 0.05) in the root lengths (44.99%), with a significant decrease (*p* < 0.05) in reactive oxygen species (ROS)-induced oxidative damage. In conclusion, *P. terrae* B6a may be a good biocontrol candidate and may be formulated into a bio-fungicide to control *F. proliferatum* and other related phytopathogens in economically important crops.

## 1 Introduction

Agriculture is a fundamental pillar of the global economy, producing both food and essential ingredients for an improved quality of life. Currently, the sector is faced with numerous challenges, including the reduction of arable land, water scarcity, the impacts of climate change, and the ineffectiveness of agrochemicals. These factors intensify both abiotic and biotic stresses on crops, alongside the increasing food demand from a growing population, ultimately leading to diminished yields (Tyagi et al., 2022). Current statistics show that by 2050, the global population is projected to reach 9.6 billion and 11 billion by 2100, necessitating a 70–100% increase in agricultural output to meet food demand (Dorling, 2021). Therefore, it is necessary to double the current food production rate and increase it by almost 50% to meet the needs of a growing population (Mittal et al., 2020). Nonetheless, the growth of agricultural land may lead to heightened deforestation, jeopardizing sustainability due to its detrimental impact on greenhouse gas emissions and biodiversity (Ioannou et al., 2020). Furthermore, the creation of stress-tolerant crop cultivars remains unavailable for commercial use. Therefore, innovative methods and strategies are continually being devised to tackle these issues.

One of the most common fungi associated with maize ear rot is *F. proliferatum* (Zhou et al., 2018). It has been linked to maize and soybean infections and has been identified as a major cause of mycotoxin contamination in food, producing a variety of mycotoxins such as fumonisins, moniliformin beauvericin, fusaproliferin, and fusaric acid (Baard et al., 2023; Lalak-Kańczugowska et al., 2023). Mycotoxins are secondary metabolites produced by pathogenic microbes with toxic effects on plants and mammals (Fandohan et al., 2003; Ridout et al., 2019). These mycotoxins cannot be removed during pasteurization, baking, cooking, and roasting (Kamle et al., 2022). Therefore, controlling Fusarium infection in crops is challenging (Khan et al., 2018).

Scientific methods for studying plant-pathogen interactions are gaining more relevance in modern agriculture, with the potential to enhance food security and eliminate malnutrition globally (Adeleke et al., 2022). Therefore, addressing these risks to the ecosystem and investigating potential endophytic bacteria will facilitate the establishment of a stable ecosystem and the cultivation of pathogen-free plants for enhanced crop production (Akanmu et al., 2021). Studies on plant-microbe interactions as bioinoculants have provided more opportunities as a substitute for chemical fungicide or pesticides usage in modern agricultural system (Orozco-Mosqueda et al., 2021; Orozco-Mosqueda et al., 2023). Interest in biocontrol of plant pathogens has significantly increased, due to the need for introduction of more environmentally friendly alternatives to the massive use of chemical pesticides (Fira et al., 2018). This method is recognized as an environmentally sustainable alternative to the extensive use of chemical pesticides (Etesami and Alikhani, 2017). The use of antagonistic microorganisms for biocontrol has been suggested as a viable alternative to agrochemicals, which have been found to have detrimental effects on the environment, especially agricultural land, as well as human health (Etesami et al., 2019). Antagonistic bacteria are viable alternatives as biocontrol agents, offering an ecologically reliable approach for protecting plants against pathogenic fungi. Therefore, the need to explore endophytic microbes as biocontrol candidates to control plant pathogens which is the focus of this study is necessary given the current challenges of food insecurity due to yield loss because of fungi infection on crops.

## 2 Materials and methods

### 2.1 Materials

The maize (*Zea mays* L. cv. 52.11R) seeds used in this study was donated by Agricol Pty Ltd., Brackenfell, South Africa. *Fusarium proliferatum* PPRI 31301 was obtained from the Plant Protection Research Institute (Agricultural Research Council, South Africa). The bacterial endophyte, isolate B6a was obtained from the Environmental Biotechnology Laboratory culture collection (University of the Western Cape, South Africa). All chemicals and reagents used in this study were purchased from reputable vendors and used according to manufacturers’ guidelines and recommendations.

### 2.2 Fungal pathogen and bacteria endophyte isolates

#### 2.2.1 Fungal pathogen

*Fusarium proliferatum* PPRI 31301 was cultured and maintained on potato dextrose agar (PDA) at 30°C for 7 days. PPRI 31301 was confirmed using ITS rDNA gene amplification previously described by Zarrin et al. (2016). Bacterial Endophyte.

The bacterial endophyte, isolate B6a was sub-cultured in Luria Bertani (LB) broth and maintained on nutrient agar at 37°C for 24 h. The identity of the B6a was confirmed using 16S rRNA gene amplification conditions as described by Baard et al. (2023).

### 2.3 Polymerase chain reaction amplicon sequencing and phylogenetic analysis

To ensures accurate verification of identity, checks for contamination, accounts for potential strain-specific variations, and validates experimental data, the amplicons of PPRI 31301 and B6a was sequenced using the BrilliantDye^™^ Terminator v3.1 Cycle Sequencing on an ABI3500xL genetic analyzer (Applied Biosystems, United States) at the Central Analytical Facilities (Stellenbosch, South Africa). The DNA sequences for both PPRI 31301 and B6a were individually edited and aligned using the Clustal W multiple sequence alignment tool in the BioEdit sequence alignment editor (version 7.2.5) to produce contiguous consensus sequences. The aligned contiguous consensus sequences of the ITS rDNA and 16S rRNA genes were analyzed using the Basic Local Alignment Search Tool (BLAST)^1^ to identify PPRI 31301 and B6a based on their highest percentage similarity. Phylogenetic analysis of the ITS rDNA and 16S rRNA gene sequences was performed independently using the Molecular Evolutionary Genetics Analysis (MEGA) software, version 11.0.13 (Kumar et al., 2018). Bootstrap analysis with 100 replicates was performed in MEGA to assess the robustness of the phylogenetic trees.

### 2.4 *In-vitro* biocontrol potential of B6a against *Fusarium proliferatum*

#### 2.4.1 Dual culture assay

The inhibitory potential of B6a against *F. proliferatum* PPRI 31301 was assessed *in vitro* using the dual-culture assay. The dual-culture assay was carried out following the protocol reported by König et al. (2024). An agar plug (5 mm in diameter) from 7-day-old actively growing mycelia of PPRI 31301was sub-cultured on fresh, sterilized PDA media. Exactly 100 μL of B6a suspension (OD_600_ = 0.5) was inoculated at four equidistant wells surrounding the PPRI 31301 colony. The control of the experiment consisted of a PPRI 31301 agar plug (5 mm in diameter) on PDA media in the absence of B6a. Each experiment was carried out in three replicates. The plates were incubated for 7 days at 25 ± 3°C, to determine the inhibitory effects of B6a. The percentage of mycelial growth inhibition (% MGI) was calculated on the last day of the experiment using the equation described by Islam et al. (2016).


(1) $$\%MGI=\left(1-\frac{Dt}{Dc}\right)\times 100$$

Where Dt = average diameter (mm) of each treatment and Dc = average diameter (mm) of the control.

#### 2.4.2 Volatile compound assay

A PDA plug (5 mm in diameter) containing fungal mycelia obtained from the edge of a 7-day-old *F. proliferatum* PPRI31301 colony was sub-cultured onto fresh PDA media whereas a single colony of B6a was spread onto Nutrient Agar. The antagonistic activity of B6a against PPRI 31301 via volatile compounds was performed using a modified method of Prakash and Arora (2021). The nutrient agar plate containing B6a was inverted over the PDA plate with PPRI 31301, and the two plates were sealed with parafilm and incubated at 25 ± 3°C for 7 days. The control consisted of a PDA plate containing an agar plug of PPRI 31301, grown under the same conditions in the absence of B6a. The experiment was performed in triplicate, with the inhibition of PPRI 31301 recorded every 24 h for 7 days. The percentage growth inhibition (% MGI) was calculated using Equation (1).

#### 2.4.3 Antifungal activity of extracellular and intracellular crude metabolites of B6a

The bacterial endophyte B6a was screened for its extracellular (ECM) and intracellular crude metabolites (ICM) production according to the method by Prakash and Arora (2021). The log phase of B6a was inoculated in LB broth and incubated at 37°C for 48 h. After incubation, the culture was centrifuged at 13,000 rpm for 15 min to separate the supernatant (extracellular metabolites) and the pellet (intracellular metabolites). To assess the antifungal activity of the metabolites, a PDA plug (5 mm in diameter) of PPRI 31301 was placed at the center of the Petri dish, and four equidistant wells were created using a sterile cork borer. A fraction of the supernatant (100 μL) or pellet (10 mg) was inoculated into each well. The plates were sealed with a parafilm and incubated at 25 ± 2°C for 7 days. The control consisted of a PDA plug of the PPRI 31301 colony in the absence of the metabolites The experiment was performed in triplicate, with the inhibition of PPRI 31301 recorded every 24 h for 7 days. The percentage growth inhibition (% MGI) was calculated using Equation (1).

### 2.5 Effect of B6a on the hyphal morphology of *Fusarium proliferatum*

The effect of the bacterial endophyte (B6a) on the morphological structure of *F. proliferatum* was assessed using high-resolution scanning electron microscopy (HRSEM) as reported by Daniel et al. (2023). The PDA plates was prepared using the dual culture method (2.4.1), and actively growing mycelia was collected and preserved in 2.5% glutaraldehyde for 12 h at 4°C. The mycelia were dehydrated in an alcohol series (30, 40, 50, 60, 70, 80, 90, and 100%) for 10 min and blotted with filter paper to remove excess ethanol before fixing onto an aluminum stub. A drop of hexamethyldisilazane (HMDS) was applied to the mycelia and allowed to dry for 45 min. The stub was covered with carbon glue and sputter coated with gold palladium alloy and placed in a scanning electron microscope for imaging.

### 2.6 Effect of B6a on the biochemical composition and enzymatic activity of *Fusarium proliferatum*

*F. proliferatum* PPRI 31301 was co-cultured with B6a in a two-way filter flask system previously described by Cordero et al. (2014), with some modifications. The bacterial endophyte B6a was cultured in 150 mL of LB broth in a 1 L filter flask sealed with a rubber stopper and incubated at 37°C for 48 h. A PDA plug (5 mm in diameter) containing fungal mycelia obtained from the edge of a 7-day-old *F. proliferatum* PPRI 31301 colony, was cultured in 150 mL of PDB in the second filter flask, also sealed with a rubber stopper. Prior to incubation, the output of the B6a filter flask was connected to the output of the PPRI 31301 filter flask through a three-way stopcock, which remained closed. To demonstrate the inhibition of PPRI 31301 growth by volatile compounds produced by B6a in a liquid medium, the three-way stopcock was opened, and the two cultures were incubated with agitation at 25 ± 2°C for 14 days. The control experiment involved culturing PPRI 31301 under identical conditions, but with the B6a filter flask replaced by a filter flask containing LB broth only. All experiments were performed in triplicate and repeated two times independently. After incubation, the PPRI 31301 culture was centrifuged at 13,000 rpm to collect the biomass and supernatant for downstream analysis.

#### 2.6.1 Estimation of extracellular and intracellular polysaccharides, and chitin contents of *Fusarium proliferatum*

The extracellular and intracellular polysaccharides and chitin contents were determined according to the protocol reported by Daniel et al. (2023). The amount of extracellular and intracellular polysaccharides contents was estimated using the Anthrone method previously described by Dhiman et al. (2022).

#### 2.6.2 Estimation cellulase and lipase activities of *Fusarium proliferatum*

Cellulase activity of *F. proliferatum* PPRI 31301 was measured using a method reported by Dhiman et al. (2022). Carboxymethyl-cellulase (CMC) was used to determine endoglucanase activity while exoglucanase activity of the crude enzyme was determined using the DNS method previously described by Dhiman et al. (2022).

Lipase activity of *F. proliferatum* PPRI 31301 was determined using the titrimetric method previously described by Dhiman et al. (2022).

### 2.7 *In planta* biocontrol potential of B6a against *Fusarium proliferatum*

#### 2.7.1 Preparation of *Fusarium proliferatum* spore suspension

*F. proliferatum* PPRI 31301 was grown on PDA media at 25 ± 2°C for 7 days. Conidial suspensions were prepared by flooding the surface of the culture pates with 7 mL dH_2_O, gently scrapping the surface with a microscope slide and filtering the suspension through autoclaved cheese cloth. The concentration of the spore suspension was adjusted to 1 × 10^6^ spores/mL by dilution and counting with a hemocytometer.

#### 2.7.2 Preparation of B6a inoculum

A single colony of B6a was inoculated into sterile LB broth and incubated at 37 ± 2°C for 24 h. The bacterial cells were harvested by centrifugation at 13,000 rpm for 10 min. The pellet was resuspended in autoclaved dH_2_O, and the optical density adjusted to 0.5 using a spectrophotometer at a wavelength of 600 nm.

#### 2.7.3 Seed sterilization

Maize seeds were incubated in a 49°C water bath for 20 min, surface sterilized in 5% sodium hypochlorite for 2 min and rinsed with sterile dH_2_O. All seeds were allowed to dry at room temperature. The experimental design consists of three treatments of 50 seeds in three replicates each. The treatment consists of control (water treatment), seeds infected only with *F. proliferatum* PPRI 31301, and seeds primed with B6a and infected with PPRI 31301. All experiments were carried out simultaneously at 25 ± 2°C with a humidity ranged between 75 and 90% day and night.

#### 2.7.4 Antagonistic effect of B6a in *Fusarium proliferatum* infected maize seeds

After sterilizing and drying the seeds under aseptic conditions, 50 seeds were bio-primed by immersing them in the B6a cell suspension with gentle agitation for 30 min. The bio-primed seeds were then air-dried in a sterile laminar flow hood for 1 h before being inoculated with *Fusarium proliferatum* PPRI 31301 spore suspension at a final concentration of 1 × 10^6^ spores/mL. The treated seeds were aseptically placed in Tupperware containers lined with moist, sterile filter paper and incubated under germination conditions (25 ± 2°C) for 7 days. Following germination, maize seeds from all treatments were harvested, and root radical length were measured using a digital caliper and ruler while root tissues were used for biochemical and enzymatic analysis.

#### 2.7.5 Measurement of superoxide content

Superoxide concentrations of maize roots in response to the different treatments were determined according to the method described by Gokul et al. (2016) with slight modification. Intact roots (0.1 g) were submerged in a solution containing; 10 mM KCN (to inhibit Cu/Zn SODs), 10 mM H_2_O_2_ (to inhibit Mn and Cu/Zn SODs), 2% (w/v) SDS (to inhibit Mn and Fe SODs), 90 μM nitro blue tetrazolium chloride (NBT) and 50 mM potassium phosphate (pH 7.0). The roots were incubated for 20 min, homogenized and centrifuged at 10,000 rpm for 5 min. The supernatant was spectrophotometrically analyzed at 600 nm and superoxide concentration was calculated using the NBT extinction coefficient of 12.8 mM cm^−1^.

#### 2.7.6 Measurement of hydrogen peroxide content

Hydrogen peroxide content was determined based on a modified method previously described by Nxele et al. (2017). Root tissues (0.1 g) for each treatment was pulverized into a fine powder using liquid nitrogen and homogenized in 10% TCA buffer. The homogenate was centrifuged at 12,000 rpm for 15 min and the supernatant was used to determine the hydrogen peroxide content. The supernatant was mixed with the reaction buffer consisting of 5 mM K_2_HPO_4_, pH 5.0 and 0.5 M KI. The samples were incubated at 25°C for 20 min and absorbance readings were recorded at 390 nm. The hydrogen peroxide content was calculated using a standard curve based on the absorbance (A390 nm) of H_2_O_2_ standards.

#### 2.7.7 Measurement of lipid peroxidation

The extend of lipid peroxidation in response to the different treatments was estimated based on the amount of malondialdehyde (MDA) measured in maize roots radicals. The MDA content was measured using the method previously described by Klein et al. (2018) with slight modifications. Root tissues (0.1 g) were homogenized in 5% (w/v) trichloroacetic acid (TCA) buffer. The extracts were then centrifuged at 13,000 rpm for 15 min. The supernatant (200 μL) was mixed with 0.5% (w/v) thiobarbituric acid (TBA, prepared in 20% TCA), followed by boiling at 100°C for 20 min. The reaction mixture was cooled immediately on ice for 10 min and the absorbance was recorded at 532 nm and 600 nm. The MDA concentration was calculated using the extinction coefficient of 155 mM^−1^ cm^− 1^.

#### 2.7.8 Determination of superoxide dismutase and ascorbate peroxidase activity

To determine total superoxide dismutase (SOD) and ascorbate peroxidase (APX) activity in maize roots, total soluble protein extracts were obtained by homogenizing 0.1 g of root tissue with extraction buffer [40 mM K_2_HPO_4_, pH 7.4, 1 mM EDTA, 5% (w/v) polyvinylpyrrolidone (PVP, molecular weight = 40,000)]. The resulting homogenates were centrifuged at 12,000 rpm for 20 min at 4°C and the supernatants were used for the spectrophotometric determination of total SOD and APX activity.

Total SOD activity of maize roots was assayed using a method previously described by Klein et al. (2018). For the spectrophotometric measurement of total APX activity in the maize roots a method of Keyster et al. (2011) was used.

#### 2.7.9 Cell viability assay using Evans blue dye

Cell viability of maize roots was assessed using a modified method previously described by Gokul et al. (2016). Root material (2 cm from tip of the root) was immersed in 0.25% (w/v) Evans blue dye and incubated at 25°C for 1 h. After incubation, the dye was discarded and the roots washed with dH_2_O for 12 h to remove any excess and unbound dye. To extract the bound dye from the dead cells, the roots were incubated in 1% (w/v) sodium dodecyl sulfate (SDS) solution at 65°C for 1 h. The extracted dye was then quantified by measuring absorbances at 600 nm using a FLUOstar Omega UV–visible spectrophotometer (BMG LabTech GmbH, Ortenberg, Germany).

### 2.8 Statistical analysis

All data generated from this study were analyzed using GraphPad Prism version 8.0.1 and SPSS version 26. Results were presented as mean ± standard error of mean (SEM) from triplicate measurements. Significance levels were determined using one-way analysis of variance (ANOVA), and differences were considered significant at *p* < 0.05.

## 3 Results

### 3.1 Molecular characterization of *Fusarium proliferatum* PPRI 31301

**Figure 1:**
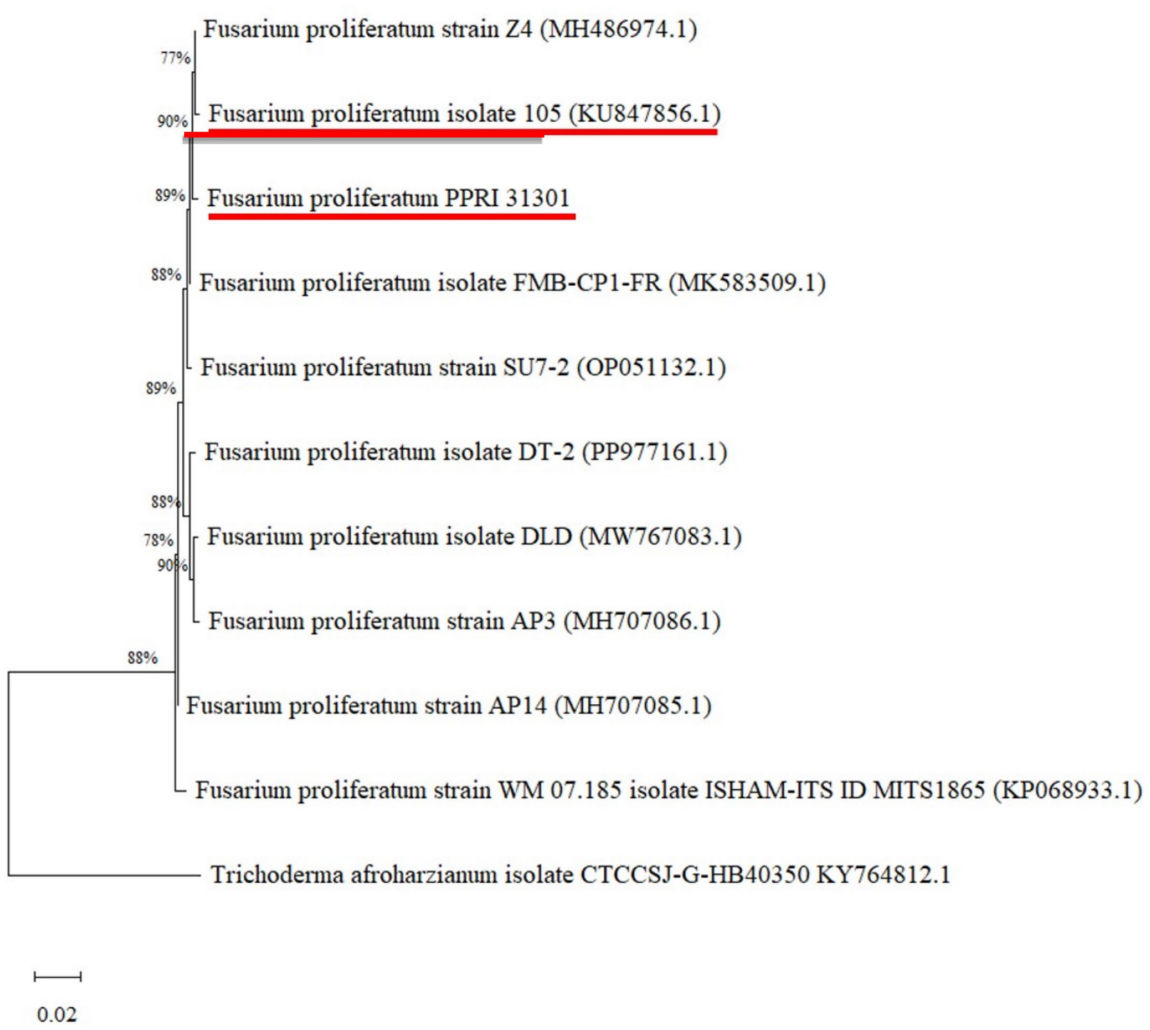
Phylogenetic analysis of ITS rDNA sequences showing the relationship between PPRI 31301 with reference sequences (with GenBank accession numbers in brackets) from NCBI using the MEGA maximum likelihood method based on the Tamura-Nei model. The bootstrap values are expressed as a percentage of 100 replicates. *Trichoderma afroharzianum* isolate CTCCSJ-GHB40350 KY764812.1 was used as an outgroup. PPRI 31301 and its closest hit are underlined in red.

The identity of PPRI 31301 was confirmed with ITS-rDNA sequencing and homology alignment using the basic local alignment search tool (BLAST) against the non-redundant NCBI database (Table 1). PPRI 31301 showed high sequence similarity (99.82%) to *F. proliferatum* isolate 105 under accession number KU847856.1. The maximum likelihood method in MEGA was used to determine the phylogenetic relationship between PPRI 31301 and selected database sequence (Figure 1).

**Table 1:** Molecular characterization of PPRI 31301 based on ITS rDNA sequencing.

Fungal pathogen ID code	NCBI GenBank accession number of the closest hit	NCBI GenBank closest hit	Similarity (%)	Completeness (%)
PPRI 31301	KU847857.1	*Fusarium proliferatum* 105	99.82	99

### 3.2 Molecular characterization of *Peaenibacillus terrae* B6a

**Figure 2:**
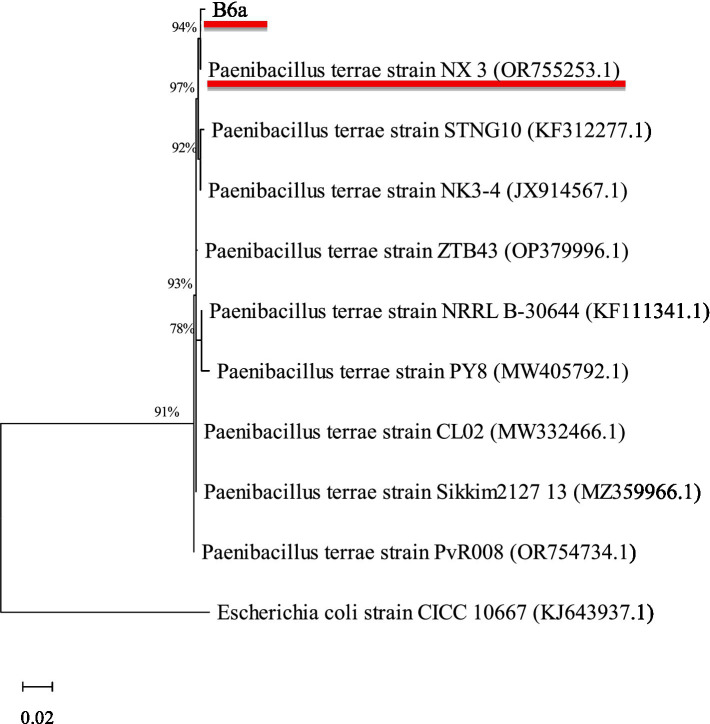
Phylogenetic analysis of 16S rRNA sequences of B6a with reference sequences (with GenBank accession numbers in brackets) from NCBI using the MEGA maximum likelihood method based on the Tamura-Nei model. The bootstrap values are expressed as a percentage of 100 replicates. *Escherichia coli* (CICC 10667) was used as an outgroup. B6a, and its closest hit are underlined in red.

Using BLAST analysis against the non-redundant NCBI database, the bacterial endophyte, B6a, showed high sequence similarity (97.81%) to *Paenibacillus terrae* NX_3 under accession number OR755253.1 (Table 2). The maximum likelihood approach was used to determine the phylogenetic relationship between B6a and selected database sequence (Figure 2).

**Table 2:** Molecular characterization of B6a by 16S rRNA gene sequencing.

Bacterial endophyte ID code	NCBI GenBank accession number of the closest hit	NCBI GenBank closest hit	Similarity (%)	Completeness (%)
B6a	OR755253.1	*Paenibacillus terrae* NX_3	97.81	94

### 3.3 *In-vitro* antagonistic effect of *Paenibacillus terrae* B6a against *Fusarium proliferatum*

**Figure 3:**
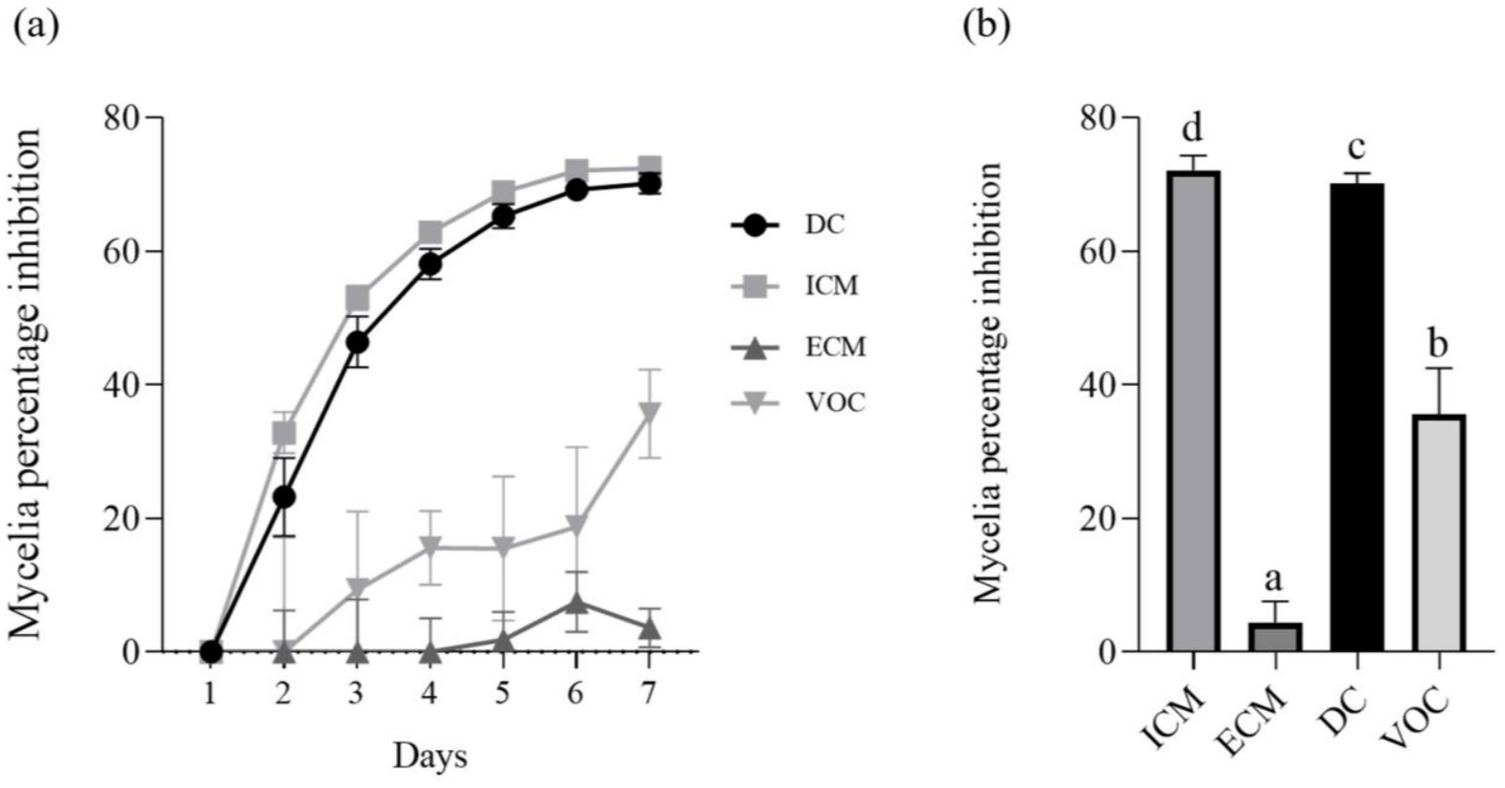
*In-vitro* antagonistic activity of *P. terrae* B6a against *F. proliferatum* **(A)** Percentage mycelia growth inhibition **(B)** Mycelia growth of *F. proliferatum* in response to *P. terrae* B6a treatment. DC, Dual culture; ICM, intracellular crude metabolite; ECM, extracellular crude metabolite; VOC, Volatile compound. Bars with different superscripts are significantly different at *p* < 0.05.

**Figure 4:**
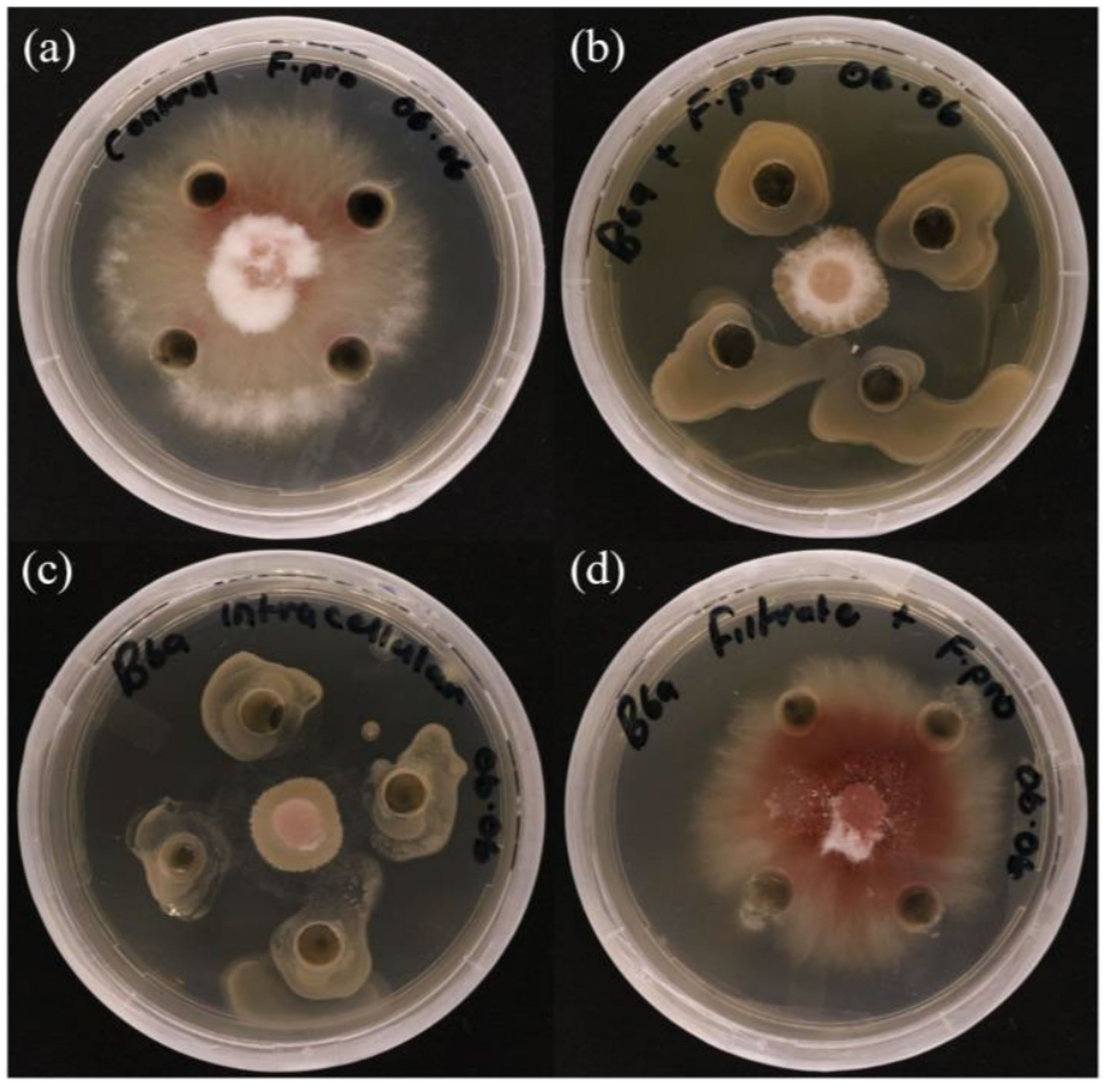
*In vitro* antagonistic activity of *P. terrae* B6a against *F. proliferatum* PPRI 31301 **(A)** Negative control. **(B)** Dual culture assay. **(C)** Intracellular crude metabolite assay. **(D)** Extracellular crude metabolite assay.

**Figure 5:**
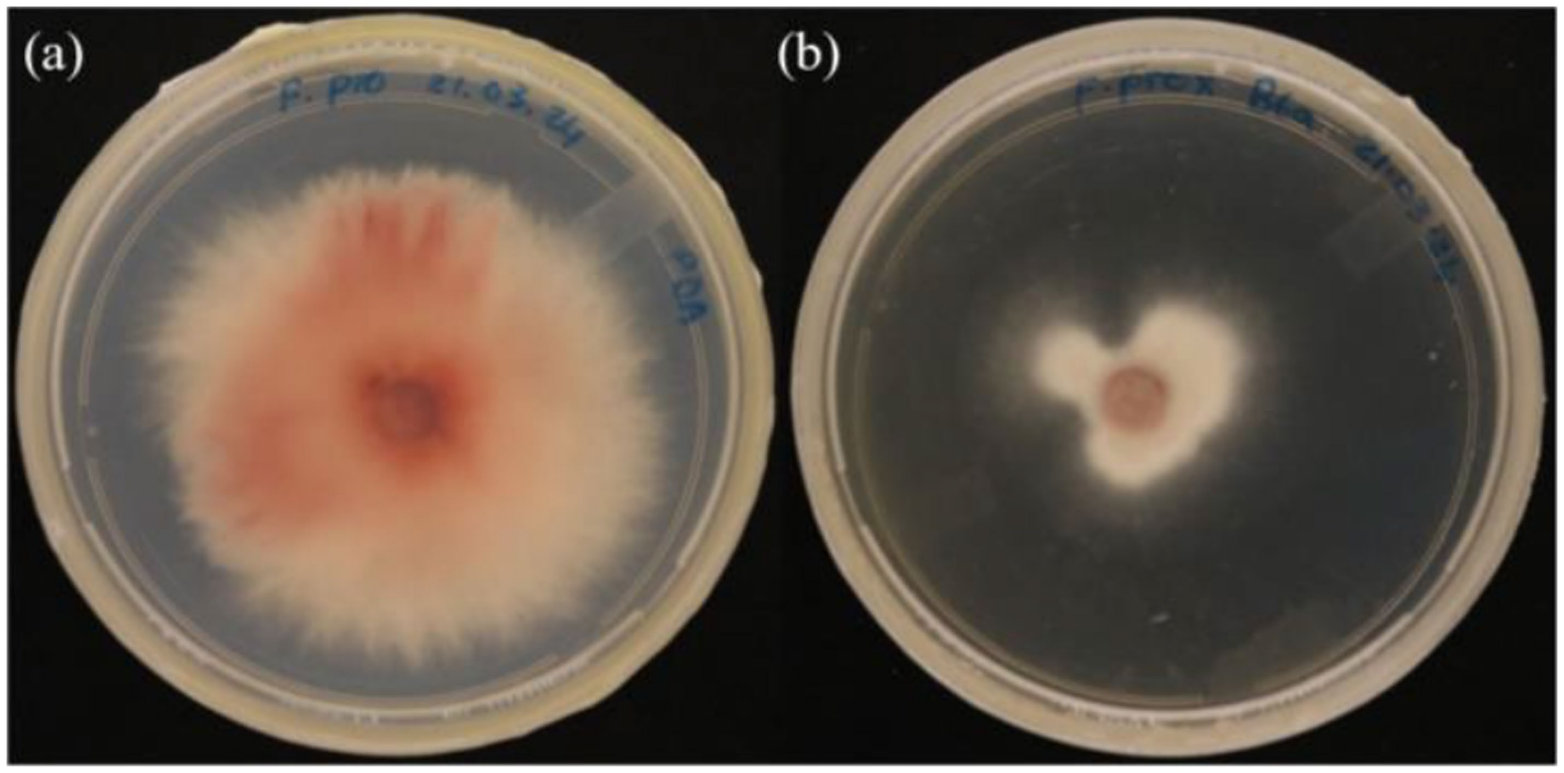
*In vitro* antagonistic assay of *P. terrae* B6a volatile compounds against *F. proliferatum* **(A)** Control **(B)** *F. proliferatum in* the presence of the volatile compounds.

The antagonistic effect of *P. terrae* B6a against *F*. *proliferatum* was evaluated. The dual culture (DC), intracellular (ICM) and extracellular (ECM) crude metabolite assays show an inhibition of 70.15, 71.64 and 3% of *F. proliferatum* mycelia, respectively (Figures 3A,B, 4A–D) while volatile compound assay shows an inhibition of 35.66% of the fungal mycelia (Figures 3A,B, 5).

### 3.4 Effect of *Paenibacillus terrae* B6a on hyphal morphology of *Fusarium proliferatum*

**Figure 6:**
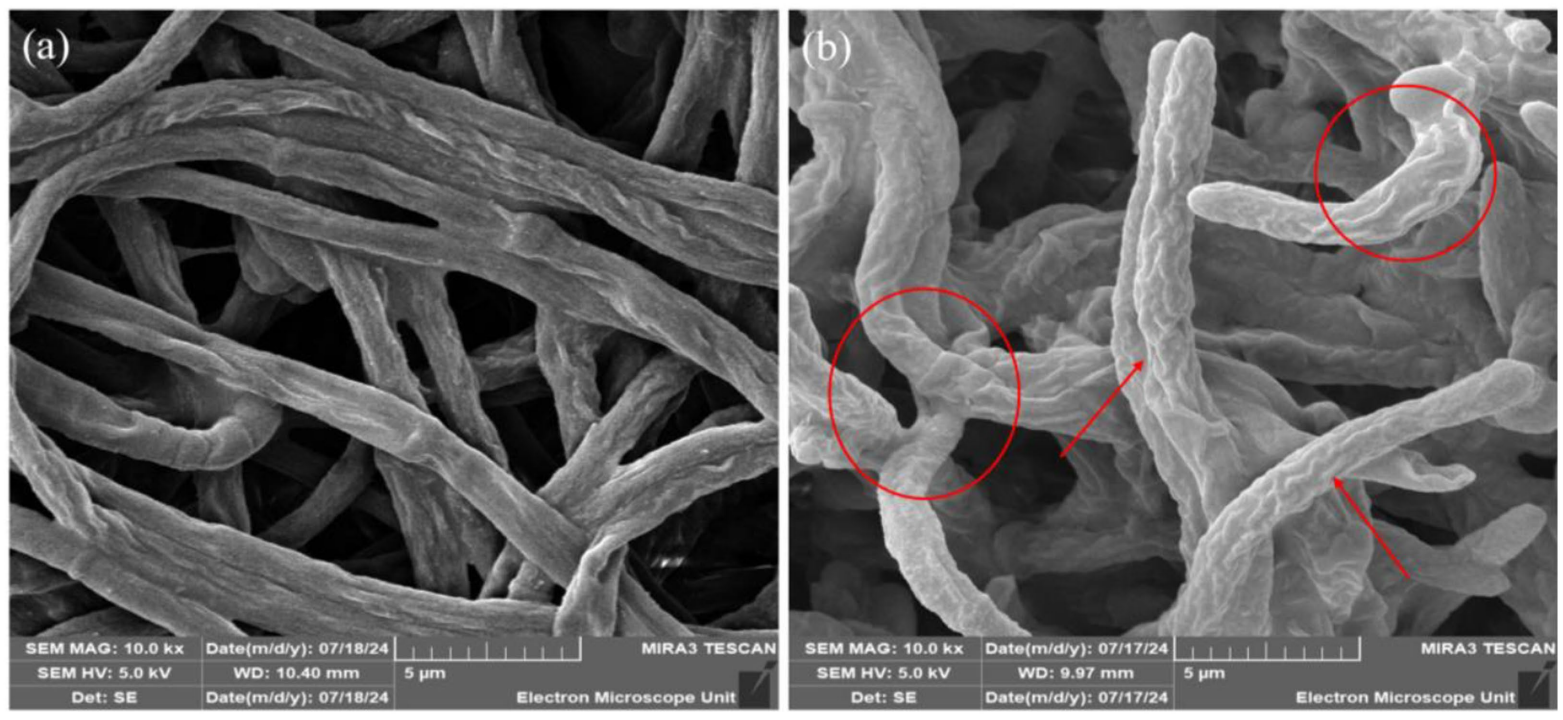
Representative SEM images of *F. proliferatum* mycelia in the presence of *P. terrae* B6a. **(A)** Control **(B)** mycelia structure in response to *P. terrae* B6a treatment. Red arrows and circles show twisting and swollen regions of *F. proliferatum* mycelia following treatment with *P. terrae* B6a.

The effects of *P. terrae* B6a on the morphology of *F. proliferatum* were examined using high resolution scanning electron microscope (HRSEM). Relative to the control, *P. terrae* B6a treatment induced significant alteration in the hyphal morphology structure of *F. proliferatum*. The treated mycelia appeared rough, twisted and swollen with moderate inflammation and the absence of septa. In contrast, the control mycelia showed normal structures with elongated, smooth surfaces and distinct septa (Figure 6).

### 3.5 Effect of *Paenibacillus terrae* B6a on polysaccharide and chitin content of *Fusarium proliferatum*

**Figure 7:**
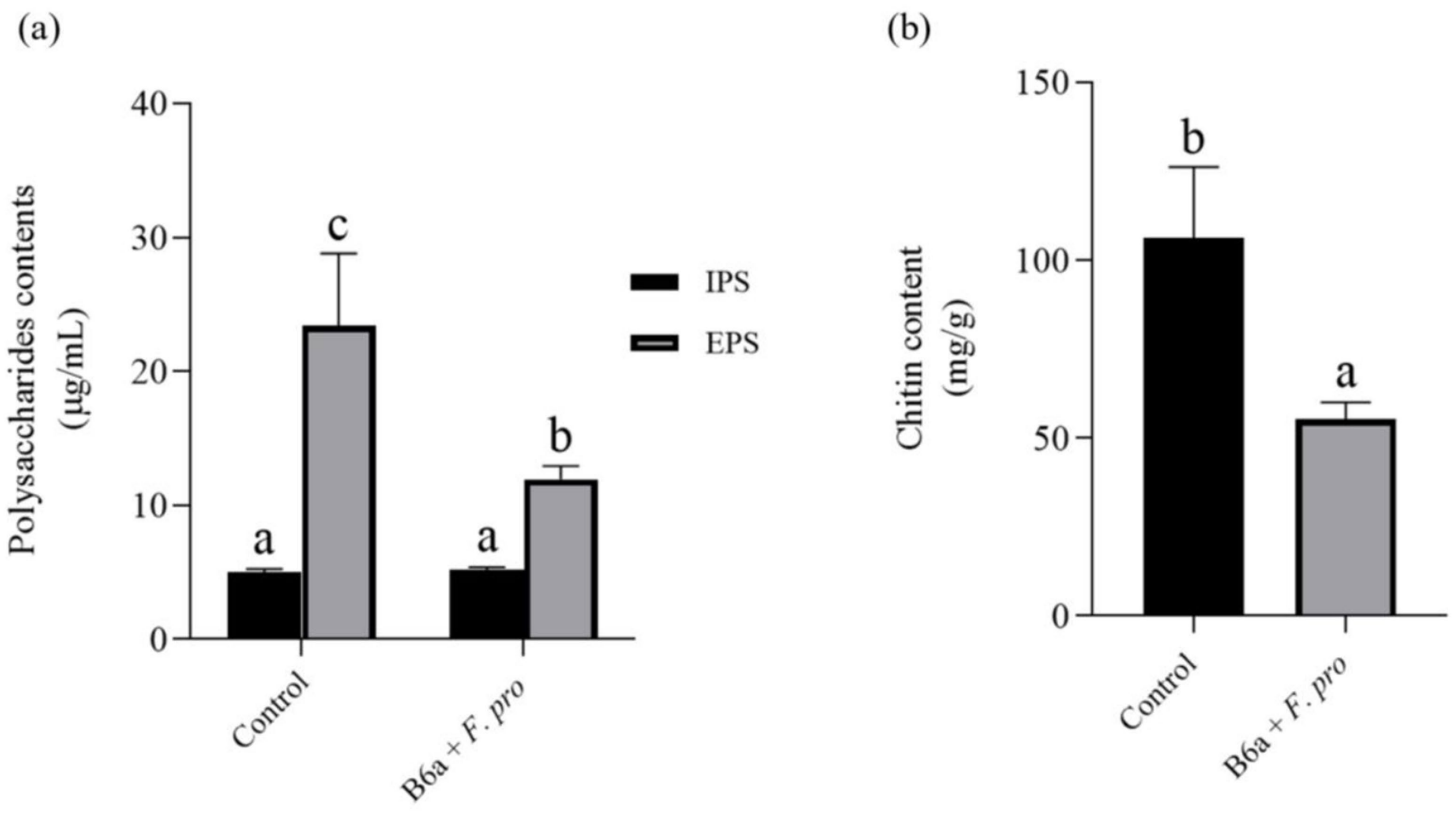
Effect of *P. terrae* B6a on **(A)** intracellular (IPS) and extracellular polysaccharide (EPS) and **(B)** chitin content of *F. proliferatum*. Bars with different alphabet are significantly different at *p* < 0.05.

The intracellular polysaccharide content of *F. proliferatum* treated with *P. terrae* B6a was not significantly different from the control (*p* > 0.05). However, the extracellular polysaccharide content showed a significant reduction of 48.99% (*p* < 0.05) compared to the control (Figure 7A). Chitin content decreased by 48.03% (*p* < 0.05), compared to the control (Figure 7B).

#### 3.5.1 Effect of *Paenibacillus terrae* B6a on cellulase and lipase activity

**Figure 8:**
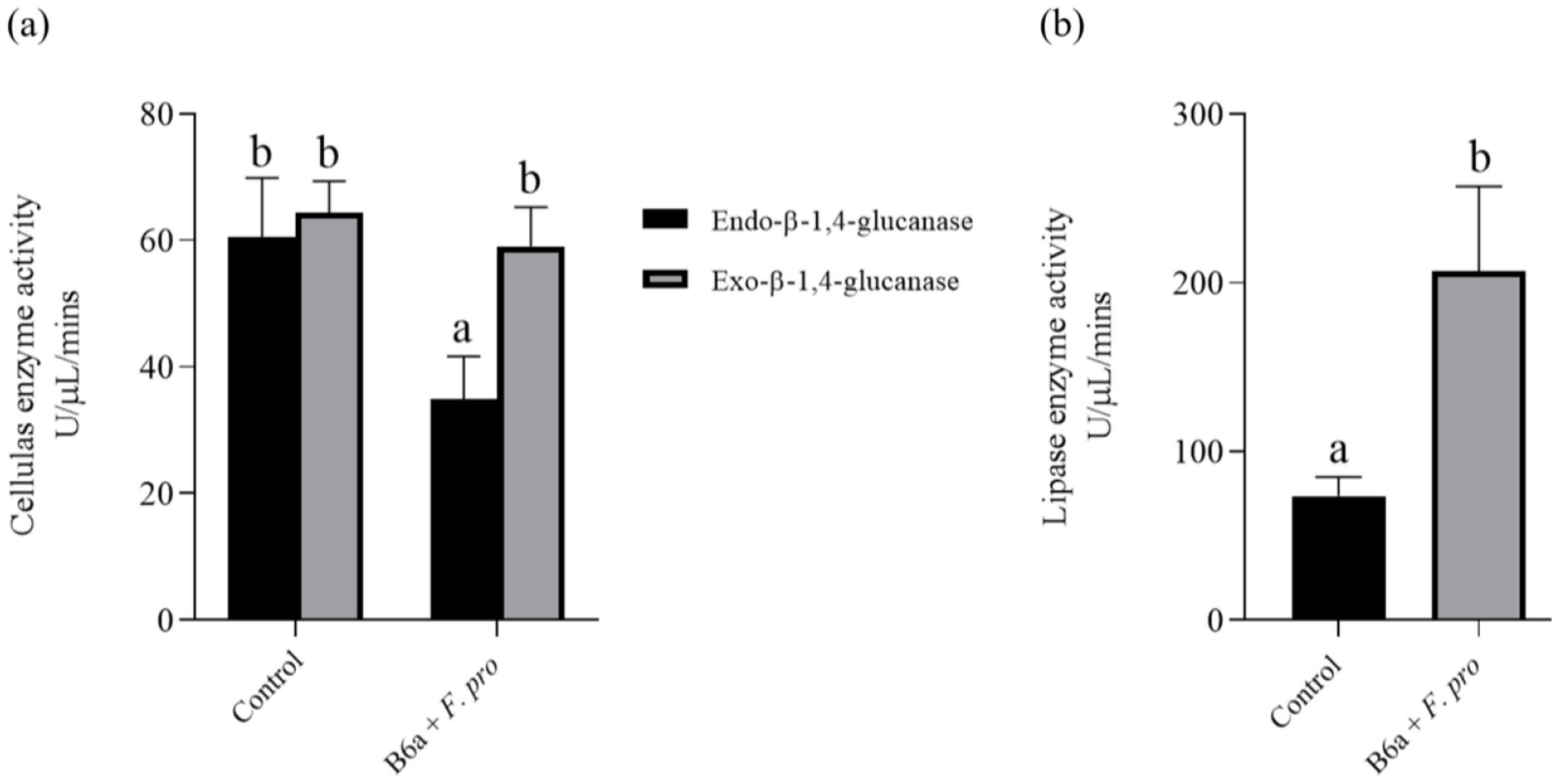
Effects of *P. terrae* B6a treatment on **(A)** endo-β-1,4-glucanase and exo-β-1,4-glucanase and **(B)** lipase activity of *F. proliferatum*. Bars with different alphabet are significantly different at *p* < 0.05.

The enzymatic activity result shows a significant decrease (*p* < 0.05) of 42.32% in the endo-β-1,4-glucanase activity of the pathogen treated with *P. terrae* B6a while no significant changes was observed in exo-β-1,4-glucanase activity of the treated pathogen relative to the control (Figure 8A). Also, there was a significant difference (*p* < 0.05) in lipase activity of *F. proliferatum* PPRI 31301which showed an increase of 64.52% when compared to the control sample (Figure 8B).

### 3.6 Effect of *Paenibacillus terrae* B6a bio-priming on maize root radical growth affected by *Fusarium proliferatum* infection

**Figure 9:**
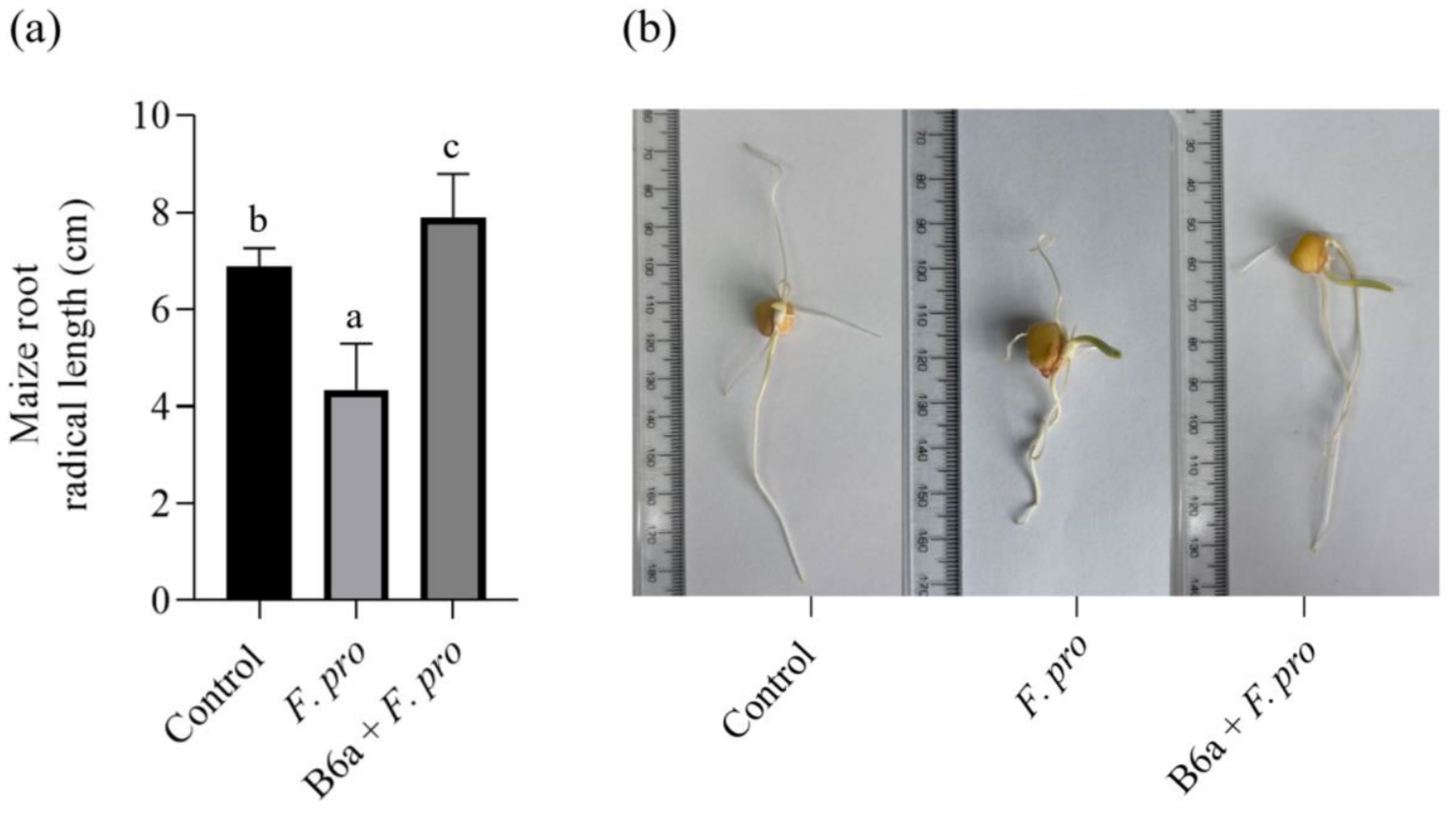
Effect *P. terrae* B6a bio-priming on root length of maize seeds infected with *F. proliferatum*. **(A)** Average root length of maize seeds primed with *P. terrae* B6a bacterial endophyte and infected with *F. proliferatum*. **(B)** Root length of maize seeds primed with *P. terrae* B6a and infected with *F. proliferatum*. Seeds were germinated for 7 days’ post priming/inoculation. Bars with different alphabet are significantly different at *p* < 0.05.

The inoculation of maize seeds with *P. terrae* B6a was assessed using the seed soaking method. Maize seeds inoculated with *F. proliferatum* PPRI 31301 shows reduction in maize root growth (Figures 9A,B). In comparison to the control with an average root length (6.89 cm), when maize seeds were infected with 10^6^ spores/mL of PPRI 31301, there was a significant decrease in root length of 37%. On the other hand, bio-priming of the infected seeds with *P. terrae* B6a shows significant recovery and increase to about 44.99% in root length compared with the infected and not treated seeds and about 12.67% improvement in root length when compared with the control seeds. Furthermore, the root of the bio-primed seeds looks thicker compared to the control and the infected seeds (Figures 9A,B).

### 3.7 Impact of *Fusarium proliferatum* infection and *Paenibacillus terrae* B6a bio-priming on reactive oxygen species accumulation in maize roots

**Figure 10:**
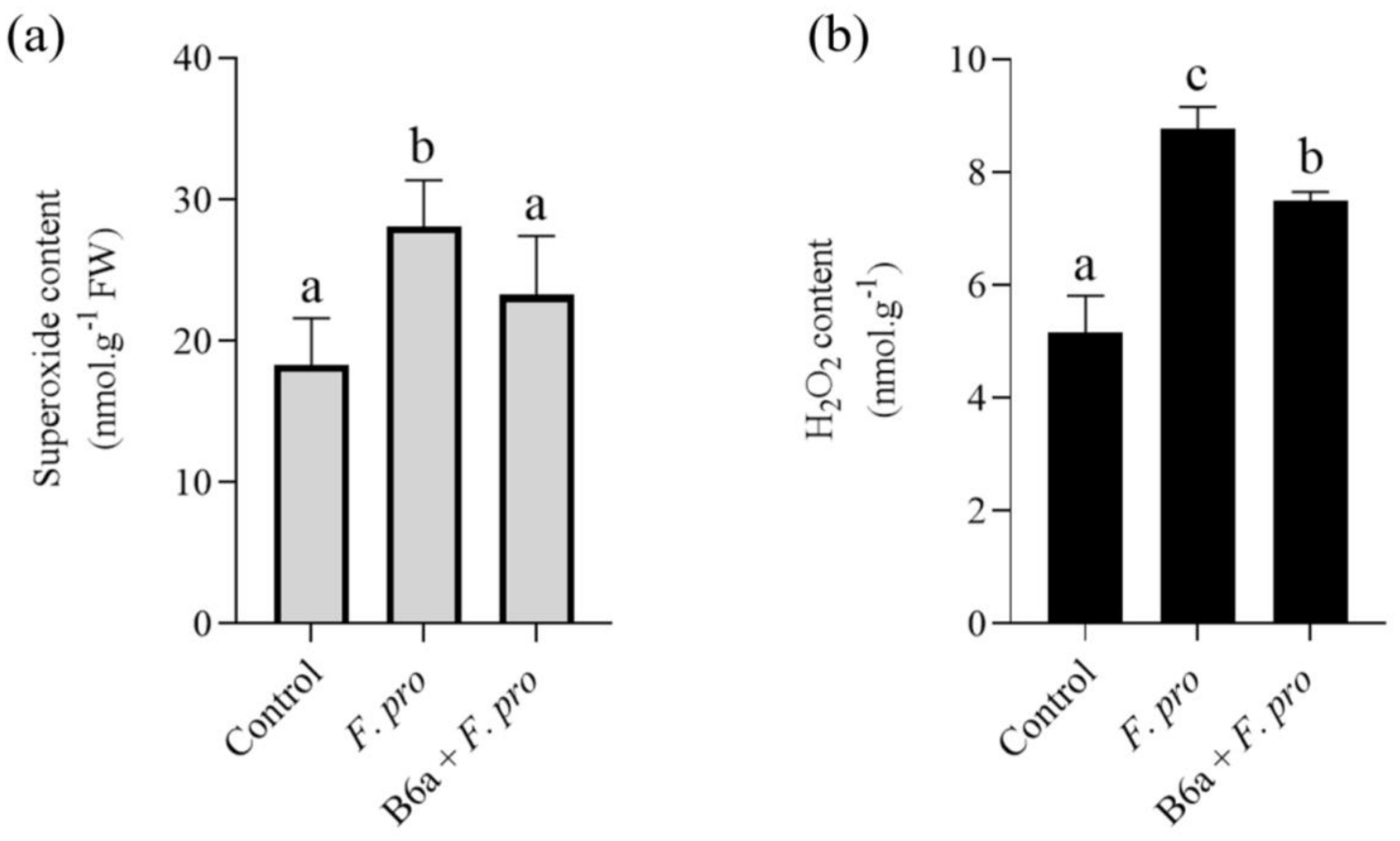
Effect of *F. proliferatum* infection on **(A)** superoxide and **(B)** hydrogen peroxide contents of maize seeds primed with *P. terrae* B6a. Bars with different alphabets are significantly different at *p* < 0.05.

The effect of *F. proliferatum* PPRI 31301 infection on induced ROS accumulation in maize roots bio-primed with B6a was evaluated after 7 days post-infection. The superoxide content in roots of maize seeds infected with PPRI 31301alone was significantly higher (*p* < 0.05) when compared to the untreated control (Figure 10A). However, in seeds bio-primed with *P. terrae* B6a and infected with PPRI 31301, there was a significant decrease (*p* < 0.05) in superoxide contents of the roots relative to the control and seeds infected with PPRI 31301alone (Figure 10A).

On the other hand, there was a significant increase (*p* < 0.05) in the hydrogen peroxide contents in roots of maize seeds infected with PPRI 31301alone compared with the controlled experiment. In seeds bio-primed with *P. terrae* B6a and subsequently infected with PPRI 31301, there was a significant decrease (*p* < 0.05) in hydrogen peroxide contents of the roots compared with the seeds infected with PPRI 31301 alone with a slight increase when compared to the controlled experiment (Figure 10B).

### 3.8 Effect of *Paenibacillus terrae* B6a bio-priming on malondialdehyde levels in maize roots infected with *Fusarium proliferatum*

**Figure 11:**
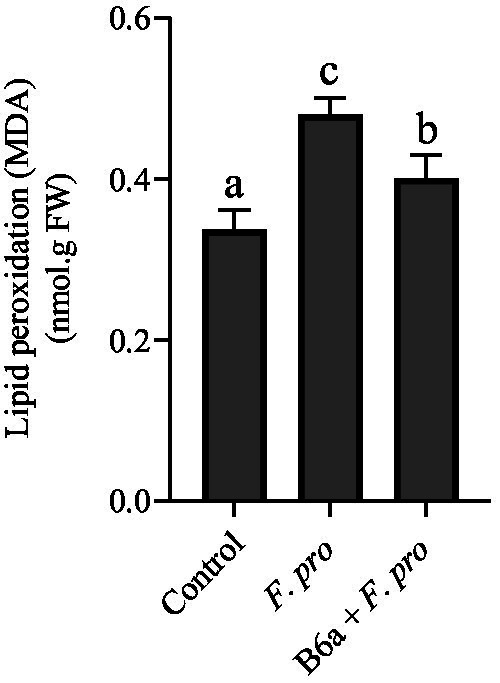
Effect of *F. proliferatum* infection on the MDA contents of maize roots primed with *P. terrae* B6a. Bars with different alphabet are significantly different at *p* < 0.05.

The MDA contents of 7-day old maize root whose seeds were primed with *P. terrae* B6a and infected with *F. proliferatum* PPRI 31301 were evaluated (Figure 11). Maize roots that were infected with PPRI 31301show a significant increase (*p* < 0.05) in MDA content (41.18%) when compared to the control experiment. In roots of maize seeds primed with *P. terrae* B6a, the MDA contents increased by 17.65% relative to the control but decreases by 20% when compared to the MDA contents of maize roots whose seeds were infected with PPRI 31301alone (Figure 11).

### 3.9 Effect of *Paenibacillus terrae* B6a bio-priming on maize roots total superoxide dismutase activity under *Fusarium proliferatum* infection

**Figure 12:**
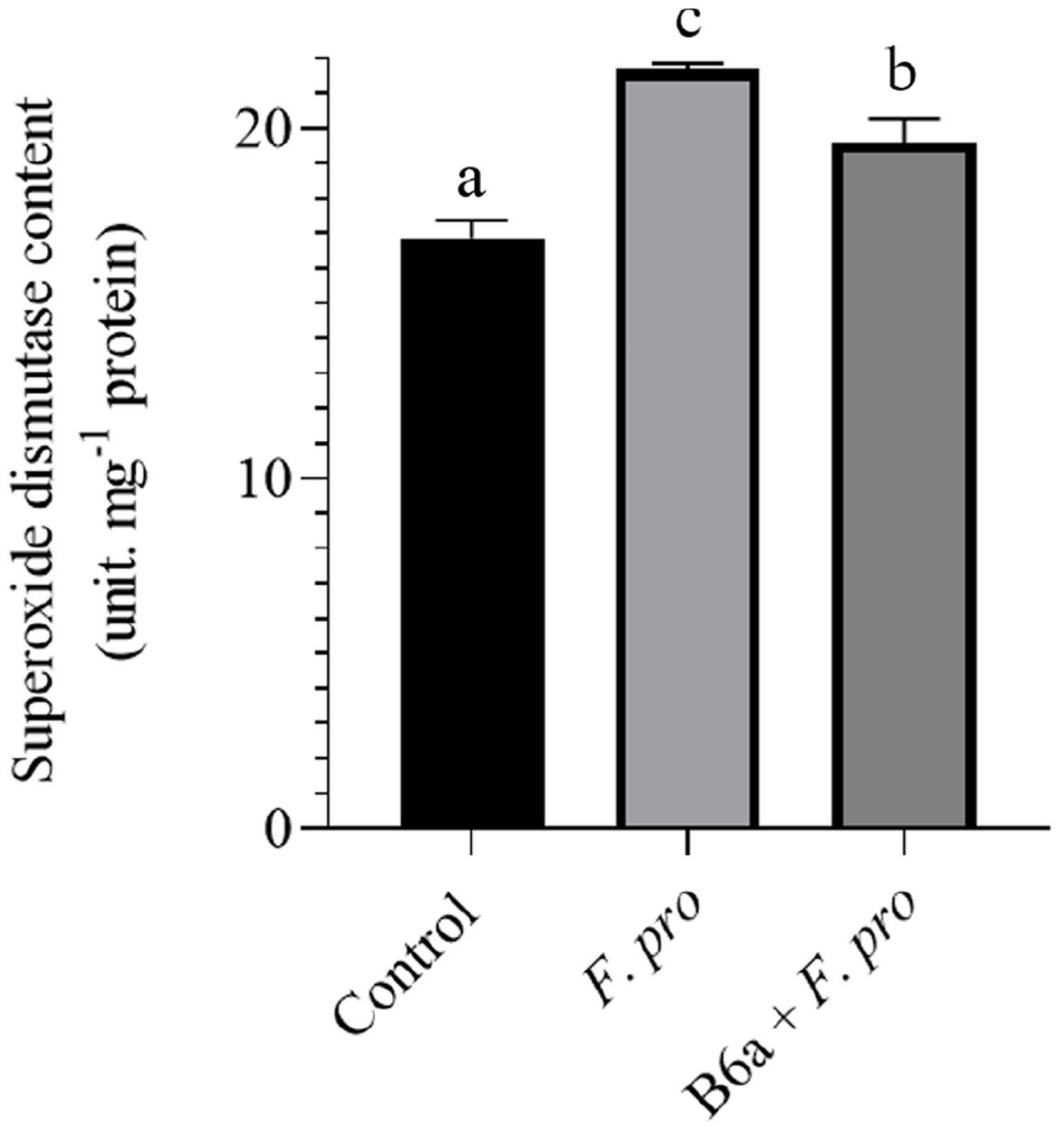
Effect of *P. terrae* B6a bio-priming on maize roots total superoxide dismutase activity under *F. proliferatum* infection. Bars with different alphabet are significantly different at *p* < 0.05.

The effect of *P. terrae* B6a bio-priming on the SOD activity of maize roots infected with *F. proliferatum* PPRI 31301 is presented below. There was a significant increase (*p* < 0.05) in SOD activity of maize roots infected with PPRI 31301alone relative to the control experiment. In the seeds primed with B6a and infected with PPRI 31301, there was a significant increase in SOD activity of the roots compared to the control but was slightly lowered compared to the roots of seeds infected with PPRI 31301alone (Figure 12).

### 3.10 Effect of *Paenibacillus terrae* B6a bio-priming on maize roots ascorbate peroxidase activity under *Fusarium proliferatum* infection

**Figure 13:**
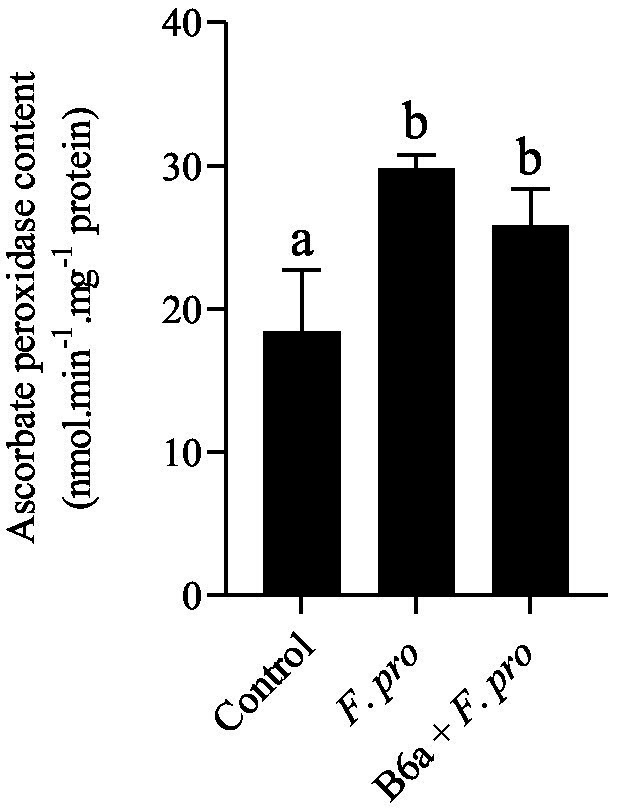
Effect of *P. terrae* B6a bio-priming on maize roots ascorbate peroxidase (APX) activity under *F*. *proliferatum* infection. Bars with different alphabet are significantly different at *p* < 0.05.

APX activity in roots of maize seeds infected with *F. proliferatum* PPRI 31301 was evaluated after 7 days post infection. The result shows a significant increase in APX activity roots of maize seeds infected with PPRI 31301 alone and roots of maize seeds primed with *P. terrae* B6a and infected with *F. proliferatum* when compared with the control experiment (Figure 13). However, APX activity in roots of maize seeds primed with *P. terrae* B6a and infected with PPRI 31301 was slightly lower compared with the roots of maize seed that were infected with PPRI 31301alone (Figure 13).

### 3.11 Effect of *Paenibacillus terrae* B6a bio-priming on cell death in maize roots under *Fusarium proliferatum* infection

**Figure 14:**
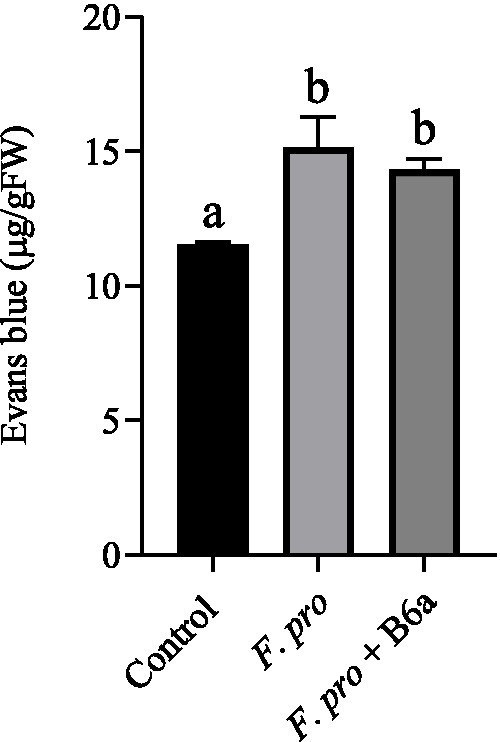
Effect of *P. terrae* B6a bio-priming on cell death in maize roots under *F. proliferatum* infection. Bars with different alphabet are significantly different at *p* < 0.05.

The effect of *F. proliferatum* PPRI 31301 infection on induced cell death of maize roots was assessed after 7 days post infection. There was a significant increase in cell death of maize roots infected with PPRI 31301 (30.31%) alone and those primed with *P. terrae* B6a and infected with PPRI 31301 (24.29%). In the control experiment, the amount of cell death was lower compared to the two treatment which is an indication that the plant is under abiotic stress due to bio-priming and infection. A slight recovery of about 5.65% in cell viability was achieved in seeds primed with *P. terrae* B6a and subsequently infected with PPRI 31301when compared to PPRI 31301 infected alone (Figure 14).

## 4 Discussion

Maize is highly susceptible to a wide range of pathogenic agents, particularly *F. proliferatum* that causes diseases such as root rot, stalk rot leading to reduced growth thereby affecting their yield (Grote et al., 2021; Krnjaja et al., 2017). Thus, biocontrol method using antagonistic bacteria represents a feasible and environmentally friendly strategy to control this pathogen. In the current study, the biocontrol potential of *P. terrae* B6a against *F. proliferatum* PPRI 31301 was evaluated *in vitro* and *in planta*. Phylogenetic analysis shows that PPRI 31301 clustered with *F. proliferatum* isolate 105 (KU847856.1) (Table 1; Figure 1). The phylogenetic analysis of *P. terrae* B6a against reference sequences from the NCBI database shows close association with *Paenibacillus terrae* NX_3 with a similarity score of 97.81% (Table 2; Figure 2).

*In vitro* antagonistic activity of *P. terrae* B6a against *F. proliferatum* PPRI 31301 shows that the *P. terrae* B6a significantly (*p* < 0.05) inhibit the growth of PPRI 31301 (Figures 3–5). During antagonistic interaction, various mechanisms such as competition for nutrient and space and synthesis diffusible compounds with antimicrobial properties are employed by bacteria to suppress or inhibit the growth of pathogens (König et al., 2024; Passari et al., 2017). From this study, PPRI 31301is more susceptible to intracellular metabolites, the dual culture and volatile organic compounds from the bacteria with an inhibition of 71.64, 70.15, and 45.83%, respectively (Figures 3–5). The pathogen was less susceptible to the extracellular crude metabolites from *P. terrae* B6a with 3% inhibition of the mycelial growth (Figures 3, 4D). The findings from this study are consistent with the report by Lim et al. (2017) and Etesami et al. (2023) who stated that the volatile organic compounds from *B. velezensis* G341 inhibit the mycelial growth of various pathogens; however, these compounds did not inhibit *Fusarium oxysporum* f. sp. lycopersici. The synthesis and activity of antimicrobial compounds such as volatile organic compounds produced by any biocontrol agents depends on the association or interactions, they are involved in which can influence pathogen susceptibility to the secondary metabolites produced (König et al., 2024; Tyc et al., 2017). For example, *P. terrae* AY-38 has been reported to produce glucanases which suppress the growth of *B. cinerea* (Lim et al., 2017). Another study shows that tridecaptin A produced by *P. terrae* NRRL B-30644 inhibits the growth of *Campylobacter jejuni* (van Belkum et al., 2015). Furthermore, Mardiah (2023) reported that the antifungal activity of endophytic bacteria, *Bacillus cereus* is attributed to the chitinase as well as other secondary metabolites produced by the organism.

To fully understand the mode of action of the antifungal activity by *P. terrae* B6a, it is important to know its impact on the structure of the pathogen. The primary point of attack of any antifungal agents or compounds is the cell wall (Daniel et al., 2024). From this study, HR-SEM analysis shows that relative to the control, the mycelia of *F. proliferatum* PPRI 31301 was distorted due to the presence of *P. terrae* B6a resulting in distortion, inflammation, twisting, and swelling of the mycelia with complete disappearance of their septa indicating a change in their cellular integrity (Figure 6). These structural changes are indicative of the antifungal activity of *P. terrae* B6a, suggesting that it effectively compromises the structural integrity of PPRI 31301, thereby inhibiting its growth. This result agreed with report by Hasan et al. (2020) who stated that endophytic bacteria produce microbial lipopeptides that lead to structural deformities in fungal hyphae, including curling, pore formation, and plasmolysis, ultimately resulting in the degradation of the hyphal structure. This indicates that the proliferation and virulence of fungal diseases can be inhibited by the presence of bacterial endophytes, which can significantly damage the integrity of fungal pathogens.

To consolidate on the antifungal activity of the *P. terrae* B6a, biochemical and enzymatic activities of *F. proliferatum* PPRI 31301 following *P. terrae* B6a treatment was evaluated. Exposure to *P. terrae* B6a to PPRI 31301 resulted in a significant (*p* < 0.05) decrease in the polysaccharide and chitin content (Figures 7A,B) and an increase in cellulase and lipase activity of PPRI 31301relative to the untreated control (Figures 8A,B). Polysaccharides and chitin are the major building blocks of fungi cell walls. A decrease in these molecules implies that the pathogen is under stress and losing its defense structure, making them susceptible to antimicrobial agents (Dhiman et al., 2022). This report also agreed with the report of Daniel et al. (2024) who reported a significant decrease in chitin and polysaccharide content of *Alternaria alternata* following treatment with antifungal agents. The decrease in polysaccharide and chitin content was further supported by the significant decrease in the exo-β-1,4-glucanase activity of PPRI 31301 in response to *P. terrae* B6a treatment when compared with the control. Glucanase hydrolysis of the internal structure of β-1,4-glucan linkages to release glucose and other fermentable sugars. The activities of these enzymes increase in the presence of abundant substrate, therefore, a decrease in their activities as observed in this study may be attributed to decrease the polysaccharide and chitin content of the organism.

Hydrolytic enzymes are secreted by pathogenic microbes to enable them to breach and invade host tissues (Mba and Nweze, 2020; Freimoser et al., 2019; Park et al., 2013). However, the roles of lipases and phospholipases in pathogen virulence remain widely unexplored. Lipolytic enzymes have also been implicated in the virulence of fungal pathogens; the contribution of lipases in fungal pathogenesis has been extensively characterized in *Candida* spp. *C. albicans* possesses at least 10 lipase-encoding genes, the expression of which is largely influenced by the stage of infection (Lopes and Lionakis, 2022; Hube et al., 2000; Park et al., 2013). Therefore, the increase in these enzymes may be a response of *F. proliferatum* PPRI 31301 to *P. terrae* B6a treatment, to defend itself against the antagonist.

The biocontrol potential of *P. terrae* B6a was also evaluated *in planta*. Maize seeds were primed with *P. terrae* B6a and infected with *F. proliferatum* PPRI 31301. Priming of the seeds with *P. terrae* B6a and subsequent infection with PPRI 31301 shows a significant improvement in the root length of the plant compared with the pathogen treatment (Figures 9A,B). This may be due to the ability of the bacteria endophyte to suppress or inhibit the growth of the pathogen which was consistent with what was observed in the *in vitro* study. According to König et al. (2024), seed priming with bacterial endophyte significantly increases the growth parameters of plants such as plant height, shoot fresh weight, and root length. This may be due to the ability of the bacteria to colonize the plant and prevent it from pathogen attack or infestation. Furthermore, Shao et al. (2023) shows that treatment of tomato plants with *B. subtilis* b2 reduced the severity of the symptoms caused by *Alternaria solani* by increasing plant height, shoot fresh weight, shoot dry weight, and root length. The authors concluded that *B. subtilis* could preserve plant biomass in infected tomato plants.

Cell death is the most effective assay for illustrating stress-induced plant damage. The production of ROS, which is part of activation of plant defense mechanisms plays an important role in limiting pathogen spread and promoting recovery (Zhu et al., 2022). From this study, there was a significant increased ROS levels including H_2_O_2_ and O_2_^−^ content in roots of maize seeds infected with *F. proliferatum* PPRI 31301 which was reduced when the seeds were primed with *P. terrae* B6a before infecting with the pathogen (Figures 10A,B). This result supports the findings by Ferrigo et al. (2021) and Lanubile et al. (2015) who reported a significant increase in H_2_O_2_ and O_2_^−^ following pathogen infection in plants. However, this effect was alleviated following priming with bacterial endophytes as reported by Mugiastuti et al. (2020) and (Chang et al., 2020).

In this study it was shown that MDA content was significantly increased due to *F. proliferatum* PPRI 31301 infection relative to the control. A slight decrease was observed in maize seeds primed with *P. terrae* B6a and infected with PPRI 31301 (Figure 11). This shows that *P. terrae* B6a mitigates the effects of *F. proliferatum*, alleviating oxidative stress. This result agrees with the report of Gupta et al. (2013) that shows *Fusarium oxysporum* infection in chickpea roots resulted in significant lipid peroxidation, correlating with elevated MDA accumulation and membrane damage. Similarly, Xie et al. (2023) and Waśkiewicz et al. (2022) have reported the upregulation of antioxidant enzymes in maize as a defense mechanism against oxidative damage in response to *F. proliferatum* infection. This implies that although increased lipid peroxidation is harmful, it also signals the plant’s defense mechanisms to be activated. The extent of lipid peroxidation and overall plant health heavily depend on the balance between ROS production and the antioxidant response (Pfordt et al., 2020; Xie et al., 2023).

The dismutation of superoxide radicals into hydrogen peroxide (H_2_O_2_) and molecular oxygen are catalyzed by superoxide dismutase (SOD), which serves as a crucial antioxidant that mitigates oxidative stress induced by pathogens (Lehmann et al., 2015; Li et al., 2015). Hydrogen peroxide (H_2_O_2_) accumulation, a byproduct of SOD activity, is not only as result of oxidative stress but acts as a signaling molecule that triggers defense responses in plants (Lehmann et al., 2015). In this study, we observed an increase in superoxide dismutase (SOD) activity in *F. proliferatum* infected maize seeds as well as those primed with *P. terrae* B6a and infected with PPRI 31301. The increase in SOD activity for the experimental treatments was significantly higher (*p* < 0.05) when compared to the untreated control (Figure 12). It is worth noting that the SOD activity observed in *P. terrae* B6a primed seeds prior to infection was lower compared to *F. proliferatum* infected seeds albeit not to the level observed in the control treatment s. Studies have demonstrated that as part of the plant’s innate immune response, a variety of pathogens can induce significant increases in SOD activity. According to Meena et al. (2016), *Alternaria alternata* infection of tomato plants resulted in increased SOD levels, which also correlated with increased MDA content, indicating increased oxidative stress. Similar trends were observed in this study, where pathogen infection resulted in elevated SOD and MDA levels. It had been reported that *F. oxysporum* infections induce SOD activity in host plants, which is important for controlling the oxidative stress associated with the infection (Wang et al., 2021). SOD upregulation during fungal infections is crucial for maintaining cellular integrity and function. Endophytic *Bacillus* and *Pseudomonas* spp. have been reported to enhance systemic resistance against pathogens (Hasan et al., 2020; Tamošiūnė et al., 2018). Fungal infections result in the production of superoxide radicals which can be detoxified by SOD, a defense-related protein produced by these endophytic bacteria (Hasan et al., 2020; Tamošiūnė et al., 2018).

Ascorbate peroxidase (APX) is an important antioxidant enzyme in plant defenses against fungal pathogen attacks that induce oxidative stress. Ascorbate peroxidase is crucial in the detoxification process of H_2_O_2_, thereby protecting plant cells from oxidative damage (Hasanuzzaman et al., 2020). The activity of APX is essential for preventing the detrimental effects of ROS on cellular components and maintaining cellular redox balance, including proteins, lipids and nucleic acids (Hasanuzzaman et al., 2020; Sadak et al., 2017). A significant increase (*p* < 0.05) in APX activity was observed in roots of maize seeds infected with *F. proliferatum* relative to the control experiment. A similar response was observed in maize seeds primed with *P. terrae* B6a and infected with PPRI 31301 (Figure 13). Our results agree with a study by Sridhar et al. (2020) who showed that infection caused by *Fusarium graminearum* of wheat lead to an increase in the expression of peroxidases. This is crucial for the defense of plants against oxidative stress caused by ROS produced during the infection process. Increase in APX activity serves as a protective mechanism, helping plants to mitigate oxidative damage and enhancing resistance to the pathogens (Sridhar et al., 2020). Another study by Nandini et al. (2010) reported that infection by fungal pathogen *Sclerotium rolfsii* resulted in elevated level of APX activity in *Arachis hypogaea*. Naveed et al. (2022) also reported that endophytic bacteria significantly elevate the antioxidant activities in marigold plants, leading to increased APX activity. This increase is essential because APX aids in the detoxification of hydrogen peroxide, a common byproduct of oxidative stress during pathogen infections. Through the enhancement of antioxidant defense mechanisms, endophytic bacteria play a crucial role in mitigating the accumulation of ROS in plants during fungal pathogen infections hence, ensuring the general well-being and development of plants under stress.

When pathogen attack or infect plants, they alter multiple growth processes which will ultimately result in the death of the whole plant. From this study, infection of maize seeds with *F. proliferatum* resulted in significant increase in the root cell death, however, when the seeds were primed with *P. terrae* B6a and then infected with the pathogen, there was a significant decrease in the cell death of the roots (Figure 14). This also shows the ability of the bacterial endophyte to protect the plant from the effect of the pathogen. Shu et al. (2017) reported that when *F. proliferatum* infect maize, it triggers a defense response, however, a weak response to counteract the aggressive nature of *F. proliferatum* leads to further tissue damage and overall cell death.

In conclusion, this study demonstrates the potential of *P. terrae* B6a as an effective biocontrol agent against *F. proliferatum*, a major pathogen in maize cultivation. The findings revealed that *P. terrae* B6a significantly inhibits the mycelial growth of *F. proliferatum* through multiple mechanisms, including the production of intracellular and volatile bioactive compounds. High-resolution scanning electron microscopy (HR-SEM) showed structural alterations in the fungal mycelia, highlighting the pathogen’s compromised integrity. Additionally, *in planta* experiments demonstrated that bio-priming maize seeds with *P. terrae* B6a mitigates pathogen-induced stress, improving root growth and enhancing antioxidant defense systems to reduce oxidative damage. These results underscore the potential of *P. terrae* B6a as a sustainable alternative to chemical fungicides, providing a promising solution for managing fungal pathogens in economically significant food/feed crops.

## Data Availability

The datasets presented in this study can be found in online repositories. The names of the repository/repositories and accession number(s) can be found in the article.

## Footnotes

^1^http://blast.ncbi.nlm.nih.gov/; accessed on 12 April 2024.
